# Women’s experiences of specialist perinatal mental health services: a qualitative evidence synthesis

**DOI:** 10.1007/s00737-023-01338-9

**Published:** 2023-06-23

**Authors:** Emma Moran, Maria Noonan, Mas Mahady Mohamad, Pauline O’Reilly

**Affiliations:** 1 Department of Nursing and Midwifery, University of Limerick, Limerick, Ireland; 2 The Department of Nursing and Healthcare, Technological University of the Shannon, Athlone, Co Westmeath Ireland; 3 Specialist Perinatal Mental Health Services, University Maternity Hospital Limerick, Limerick, Ireland

**Keywords:** Qualitative review, Qualitative evidence synthesis, Perinatal mental health, Women’s experiences, Maternal mental illness, Perception of services

## Abstract

**Purpose:**

Specialist perinatal mental health services identify and treat women experiencing mental health conditions during pregnancy and up to one year post birth. There is limited knowledge about women’s experiences of care from specialist services. Evaluation and optimisation of service delivery requires knowledge of women’s care experiences. This review aimed to systematically identify, appraise, and synthesise qualitative evidence exploring women’s experiences of specialist perinatal mental health services.

**Methods:**

A systematic literature search of five databases: Medline (OVID), EMBASE (Elsevier), PsycINFO (EBSCO), CINAHL (EBSCO) and Scopus (Elsevier), grey literature searching, and backward citation, identified a total of 1035 papers of which sixteen met inclusion criteria. Methodological quality of the included studies was assessed using the Critical Appraisal Skills Program (CASP) tool.

**Results:**

Thematic synthesis identified three themes: connected relationships; new beginnings; and meaningful service delivery. Findings identified that relationships developed with clinicians were significant to women and their experience of care. Women valued continuity of care from dedicated non-judgemental clinicians. Peer support from other mothers was perceived as meaningful to women. Through service interventions women gained new insights into their infant’s needs and grew in confidence as a mother.

**Conclusions:**

Women require provision of flexible and accessible specialist services with clinicians who are sensitive to their individual psychosocial needs and preferences. Examining discharge practices and continuing care needs is essential to ensure the best outcomes for women and their families.

## Introduction

Poor perinatal mental health is a growing public health concern in which women present with new and recurrent mental health conditions, ranging from mild to moderate anxiety and depression to more severe presentations and diagnoses (Howard and Khalifeh 2020; Markey et al*.* 2022; Redshaw and Wynter 2022). Those identified as being at significant risk of developing a new or recurrent mental health condition during the perinatal period (pregnancy, and the first year postpartum), are often referred to specialist perinatal mental health services (PMHS). The prioritisation of women’s perinatal mental health (PMH) can be seen globally in the development of specialist services (Powell et al. 2020).

Specialist PMHS can be defined as services which focus on the prevention, detection, and management of perinatal mental health conditions in pregnancy and the first year postpartum (Howard and Khalifeh 2020; Wrigley and O'Riordan 2023). This rapidly expanding specialist area of mental health requires treating clinicians to have specific knowledge and expertise in assessment, diagnosis, and treatment of women across the perinatal period (Galbally et al. 2013). To alleviate the long-term socioeconomic costs of PMH conditions, and in recognition of significant maternal mortality rates, PMHS have now integrated mental healthcare into maternity services in many high-income countries (Howard et al. 2014; Sambrook Smith et al. 2019; Huschke et al. 2020).

Specialist PMHS are designed to optimise maternal mental health during the perinatal period and create immediate and long-term positive outcomes for women, their infant(s), and family (Mongan et al. 2021). Routine mental health screening of women through maternity services is common practice (Coates and Foureur 2019), with services aimed at providing timely assessment and treatment of PMH conditions and comorbid difficulties (Howard and Khalifeh 2020). Preconception counselling services for women with a history of pre-existing moderate or severe mental health conditions such as bipolar affective disorder, previous postnatal psychosis, or severe depression, are also provided by many specialist PMHS (Mongan et al. 2021).

Despite considerable investment in service development the provision of specialist PMHS remain variable across the world (Ganjekar et al. 2020; Schwank et al. 2020; Mongan et al. 2021). Countries with specialist PMHS include the UK (Howard and Khalifeh 2020), Australia (Centre of Perinatal Excellence (COPE) 2017), New Zealand (Ministry of Health, 2021), Ireland (Wrigley and O'Riordan 2023), the US (Schiller et al. 2020), and France (Sutter-Dallay et al. 2020). Research in low- and middle-income countries remains focused on the prevalence of PMH conditions, risk factors associated with poor PMH, and the limited access to mental health resources (Redshaw and Wynter 2022; Umuziga et al*.* 2022). Furthermore, inconsistent and inequitable service provision particularly in rural areas continues in countries with existing PMHS (Humphreys et al. 2016; Huschke et al. 2020). Mother and Baby Units (MBU) have been established in several European countries, Australia, and more recently Sri Lanka, India, the US, and New Zealand for women requiring psychiatric admission in the postnatal period (Howard and Khalifeh 2020). Women living in countries or regions without access to a MBU are separated from their baby and admitted to general psychiatric wards (Mongan et al. 2021).

Although countries with existing PMHS are experiencing rapid growth to meet the increasing demands for clinical services (Schiller et al. 2020), many system level barriers have been identified. These barriers include fragmented services, poor continuity of care, and limited pathways of care and referral options (Coates and Foureur 2019; Sambrook Smith et al. 2019; Dadi et al. 2021).

The identified system level barriers are further compounded by barriers from the women themselves who can be reluctant to disclose PMH symptoms despite regular contact with health services (Ayres et al. 2019). Women’s reluctance to disclose mental ill health is often due to fears of stigma, losing parental rights, being deemed an unfit mother, or difficulties with the logistics of attending appointments when caring for small children (Ford et al. 2019; Noonan et al. 2019).

Integral to the evaluation of specialist PMHS is an assessment of the acceptability of the provided services to the women themselves, with service user satisfaction shown to be an accurate indicator of quality in mental healthcare (Bastemeijer et al. 2019; Powell et al. 2020). National and international recognition has been given to service user engagement in shaping and informing health policy. The World Health Organisation (WHO 2016) describes service user and family engagement as essential in developing high-quality person-centred services. Calls have been made for increased focus on woman-centred research, bringing subjective experiences and knowledge of women’s PMH needs to the fore (Myors et al. 2013; Huschke et al. 2020; Lever Taylor et al. 2020). Furthermore, a systematic review by Watson et al. (2019) recommends that women's expressed needs be used in the co-production of PMH policies, interventions, and services. To our knowledge no previous qualitative evidence synthesis has been identified exploring women’s experiences of specialist PMHS. This review aimed to systematically identify, appraise, and synthesise qualitative evidence exploring women’s experiences of specialist PMHS. Findings of this review have the potential to provide a comprehensive understanding of women’s experiences of specialist PMHS which can be utilised to inform policy and practice developments.

## Methods

A qualitative evidence synthesis (QES) with thematic synthesis was chosen as the most appropriate method to address the review question. A QES is a systematic review of qualitative research evidence which enables the researchers to present a rich interpretation of the phenomenon and gain a greater appreciation of women’s experiences, ideas, and priorities (Lewin et al. 2018; Flemming et al. 2019). Thematic synthesis is one of the most commonly used methods of QES and has been used as an approach to address a wide range of research questions, particularly those which seek to explore individuals’ experiences of health care (Flemming and Noyes 2021). This review was carried out according to preferred reporting items for systematic reviews and meta-analyses PRISMA 2020 guidelines (Page et al. 2021) and Enhancing transparency in reporting the synthesis of qualitative research (ENTREQ) reporting guidelines (Tong et al. 2012) (Checklist Supplementary File 1). A protocol for this review was registered with PROSPERO (CRD42022298704). A PEO framework was used to develop the review question and establish search terms (Table 1).

**Table 1:** PEO framework

Population	women, pregnant women, female, postnatal, prenatal, antenatal, women of childbearing age, maternal, mothers, perinatal, peri-natal, childbirth, puerperal, pregnancy
Exposure	perinatal service, perinatal mental health service, specialist perinatal mental health service, mental illness, mental illness in pregnancy, mental ill health, postpartum mental illness, postnatal mental illness, perinatal mental illness, mental health treatment, perinatal mental health team, postpartum treatment, mental health treatment, health services, care and treatment, intervention, support
Outcome	experiences, engagement, treatment experiences, treatment outcomes, views and opinions, perceptions of treatment, service user views, views and opinions, perspectives, lived experience

In consultation with an information specialist librarian the search strategy for this review was developed using keywords established in the PEO framework. The final MEDLINE search string is available in Supplementary File 2. The review question was formulated being: ‘What are women’s experiences and perceptions of the care received from specialist PMHS?’. In addition, the review aimed to identify (a) what factors influenced how women perceived their care and treatment? and (b) what research gaps, if any, warrant further investigation to inform evidence-based knowledge in specialist PMHS delivery.

## Eligibility Criteria

Inclusion of primary qualitative studies exploring women’s experiences of care from a specialist PMHS during preconception, pregnancy or up to one year postpartum. Mixed methods studies presenting qualitative data were eligible for inclusion. Studies were excluded when (1) women received mental healthcare from a source other than a specialist PMHS (2) women were under 16 at the time of childbirth (3) papers were editorials, opinion papers, discussion papers, policy statements, or literature reviews and (4) published prior to January 2010.

## Search Strategy

A preliminary literature search of the Cochrane library and PROSPERO was conducted to identify that no current listed reviews have, or were being, conducted on this research topic. To test for accuracy of the established search terms a search was conducted on Medline. An electronic database search of Medline (OVID), EMBASE (Elsevier), PsycINFO (EBSCO), CINAHL Complete (EBSCO) and Scopus (Elsevier) was conducted to identify studies which met eligibility criteria. Additionally, grey literature was searched using OpenGrey and Greylit. The search strategy included the use of Boolean operators ‘AND’ and ‘OR’, free text searching, synonyms, and MeSH index terms. The literature search timeline was from January 2010 to January 2022 as this has been a time of significant investment and development of specialist PMHS. No limitation was placed on studies location or language. Hand searching of included studies reference lists was also performed.

## Quality appraisal of included studies

Methodological quality of the included studies was assessed using The Critical Appraisal Skills Programme (CASP) qualitative checklist tool (Supplementary File 3). CASP items were scored independently by the first author [EM] in consultation with the review team. No papers were excluded based on the outcome of the assessment (Long et al. 2020). The methodological quality appraisal was then used to inform the GRADE CERQual assessment of review findings.

## Data extraction and synthesis

Data reported in the ‘results’ section of included studies were extracted into a custom designed table which included participant quotes and primary author interpretations. Only data relevant to the research question exploring women’s experiences of specialist services were extracted. Thematic synthesis was guided by Thomas and Harden (2008) and involved three stages (1) line-by-line coding of data results, (2) developing descriptive themes according to the data’s meaning and content, (3) generating analytical themes. The first stage involved each line of text being read, re-read, and coded based upon its meaning and content. Each sentence had at least one code applied, and most were labelled using several codes (Thomas and Harden 2008). Consistency of interpretation was ensured by multiple authors independently reviewing the applied codes. In the second stage similarities and differences among the codes were grouped and discussed in consideration of the review question. The third stage involved 'going beyond' the findings of the primary studies using an inductive process to generate additional concepts and understandings (Thomas and Harden 2008). To ensure rigor and credibility, the research team discussed each stage of the synthesis process and agreed upon coding and themes by consensus.

## Reflexivity Statement

Reflexivity is an integral part of conducting rigorous qualitative research. Described as a process of continual internal dialogue and self-evaluation reflexivity examines researcher’s positionality and impact on the research process (Berger 2015; Dodgson 2019; Trainor and Bundon 2021). The primary author (EM) is a researcher, mother, mental health nurse, and lecturer studying in the field of PMH. This author has no personal experience of perinatal mental health difficulties but has a professional interest in women’s mental health. Reflection on positionality took place throughout the research process through reflexive note taking and frequent research team discussion.

## Results

### Study Characteristics

**Fig. 1:**
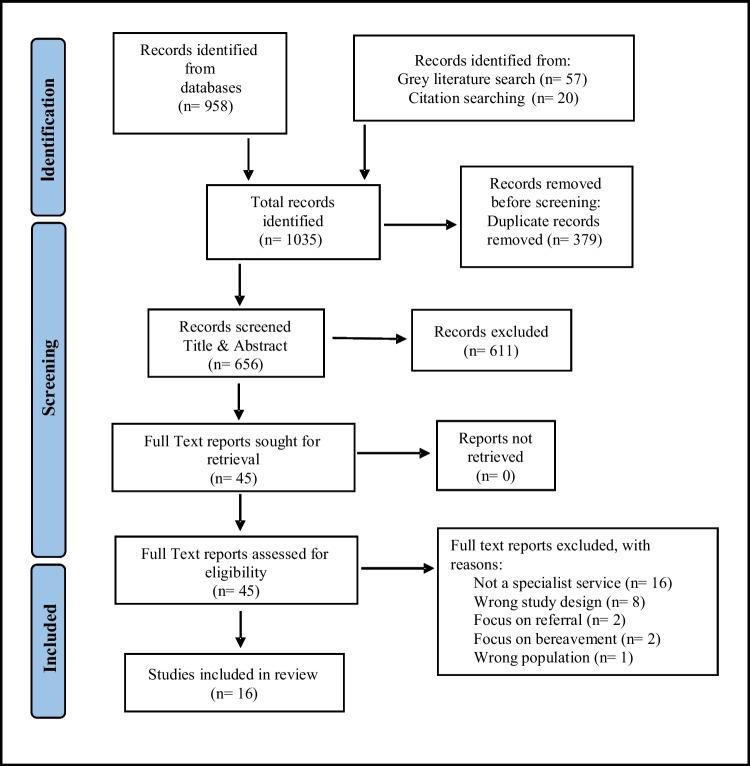
PRISMA flow diagram of study search and selection process (Page et al. 2021)

Electronic database searching identified 958 records. Grey literature searching identified 57 records. Citation searching identified 20 records (Fig.1). All 1035 records were exported to Endnote 20 and duplicates removed (n=379). A total of 656 studies remained for title and abstract screening, which excluded 611 records. Full text screening was conducted independently by two authors. Group discussion and independent reviewing by a third author resolved differing opinions on eligibility. Reasons for exclusion of full text papers were documented and reported in PRISMA flow diagram (Fig. 1). No eligible studies were identified from the grey literature records. Reference list checks of included studies yielded 4 eligible papers. A total of 16 papers, representing 12 individual studies, met inclusion criteria for this review (Fig. 1).

A summary of study characteristics is reported in Table 2. Five countries are represented in the studies of this review: United Kingdom (n=7) (Griffiths et al. 2019; Lever Taylor et al. 2019; Lever Taylor et al. 2020; Powell et al. 2020; Bruce and Hackett 2021; Greaves et al. 2021; Pilav et al. 2022); Australia (n=6) (Hauck et al. 2013; Myors et al. 2014a; Myors et al. 2014b; Myors et al. 2015a; Myors et al. 2015b; Coates et al. 2017), New Zealand (n=1) (Wright et al. 2018), Ireland (n=1) (Higgins et al. 2016), and Canada (n=1) (Viveiros and Darling 2018). Interviews were the most frequently used method for data collection used in all but one study, which performed qualitative thematic analysis on written feedback from a questionnaire (Powell et al. 2020). Of the studies eleven were qualitative and five were mixed method design. Studies reported women’s experiences of various specialist PMHS including: MBUs (Wright et al. 2018; Griffiths et al. 2019; Powell et al. 2020), perinatal and infant mental health (PIMH) services (Myors et al. 2014a; Myors et al. 2014b; Myors et al. 2015a; Myors et al. 2015b; Coates et al. 2017), PMHS (Higgins et al. 2016; Viveiros and Darling 2018), community PMHS (Lever Taylor et al. 2020), specialist dialectical behavioural therapy (DBT) and art therapy interventions (Bruce and Hackett 2021; Greaves et al. 2021), specialist childbirth and mental illness antenatal clinic (Hauck et al. 2013), experiences of service engagement with families (Lever Taylor et al. 2019), and treatment during the COVID-19 pandemic (Pilav et al. 2022). Studies were published between 2013 and 2022. Only one included study examined data on women’s PMHS experience during the COVID-19 pandemic (Pilav et al. 2022). The included studies Lever Taylor et al. 2019 and Lever Taylor et al. 2020 formed part of a wider qualitative study exploring experiences of perinatal mental healthcare in England. The included papers by Myors et al. reported separate findings from a single mixed-method study examining therapeutic interventions in specialist PIMH services (Myors et al. 2014a; Myors et al. 2014b; Myors et al. 2015a; Myors et al. 2015b).

**Table 2:** Summary of study characteristics

Author(s), Year, Title, Country	Study aims	Study design	Data collection	Study Participants	Method of analysis	Key Findings
Bruce and Hackett (2021) Developing art therapy practice within perinatal parent-infant mental health UK	Describe the practice of art therapy in PIMH alongside the views of mother’s experience and perceived outcomes	Mixed methods	Semi structured interview and two self-reporting closed question questionnaires	Nine women. Mean age 27 years, (range 17 to 37 years). Low-income households n=5 (55%).	Interview reported in vignette	Art therapy perceived as helpful. Positive changes to self-understanding, comprehension of problems, and mood.
Coates et al. (2017) The experiences of women who have accessed a perinatal and infant mental health service: a qualitative investigation Australia	Investigate the experiences of women who have accessed a perinatal infant mental health service	Informed by the principles of Qualitative grounded theory	Semi structured telephone interviews	Forty women. Demographics not reported.	Thematic analysis	Trusting relationships with clinicians facilitated a safe environment to reflect on trauma, mental health, and parenting.
Greaves et al. (2021) The impact of including babies on the effectiveness of dialectical behaviour therapy skills groups in a community perinatal service UK	Evaluate the impacts of DBT skills groups for mothers and babies in a community perinatal service	Mixed methods	Semi-structured interviews with outcome measures pre and post intervention	Twenty-seven women. Mean age 34 years, (range 26 to 45 years). Primiparas (55.6%), multiparas (44.4%). Primary diagnosis postnatal depression (n=8, 29.6%).	Thematic analysis guided by Braun and Clarke (2006)	DBT skills group significantly improved levels of psychological distress and emotional regulation.
Griffiths et al. (2019) A qualitative comparison of experiences of specialist mother and baby units versus general psychiatric wards UK	Compare qualitative experiences of mother and baby units and general psychiatric wards from the perspectives of women and clinicians	Qualitative descriptive approach	Semi structured interviews with participating women and focus groups with clinicians	Fifteen women and seventeen clinicians. Mean age 32 years, (range under 20 to 39 years). Primiparas (60%), multiparas (40%). Primary diagnosis postpartum psychosis n=8 (29.6%).	Thematic analysis guided by Braun and Clarke (2006)	MBUs provided perinatally focused, family centered care. Difficulties transitioning home after discharge and problems accessing MBU care reported.
Hauck et al. (2013) Pregnancy Experiences of Western Australian Women Attending a Specialist Childbirth and Mental Illness Antenatal Clinic Australia	Explore the experiences of Australian women attending a specialist childbirth and mental illness antenatal clinic including service satisfaction	Qualitative exploratory design	Telephone interviews	Forty-one women. Mean age 29.4 years, (range 19 to 40 years). Primiparas (58.5%), multiparas (41.5%). Primary diagnosis bipolar affective disorder (56.1%).	Thematic analysis	Seeing the same clinician helped women build relationships and feel understood. Women felt safe and trusted by clinicians who were respectful and empathetic.
Higgins et al. (2016) Mothers with mental health problems: Contrasting experiences of support within maternity services in the Republic of Ireland Ireland	Explore the views and experiences of women receiving care from publicly funded maternity services during pregnancy, childbirth, and immediate postnatal period	Qualitative descriptive design	In-depth face-to-face interviews	Twenty women. Mean age 33.05 years, (range 23 to 40 years). Primiparas (50%), multiparas (50%). Married n=16 (80%), co-habiting n=4 (20%).	Inductive thematic analysis	Specialist PMHS provided consistency, continuity, and dependability of care. Clinicians acted as advocates. Receiving specialist advise about medication was important to women.
Lever Taylor et al. (2019) Experiences of how services supporting women with perinatal mental health difficulties work with their families: a qualitative study in England UK	Expand on previous research by exploring the role of partners and wider family in relation to women’s perinatal mental health/ access to services and experiences of family inclusion	Qualitative	Semi-structured interviews	Fifty-two women and thirty-two family members. Mean age 32 years, (range 19 to 43 years). Primiparas (50%), multiparas (50%). Primary Diagnosis depression (37%).	Thematic analysis, guided by Braun and Clarke (2006)	Families were excluded and overlooked by services supporting women with PMH difficulties. Women who desired privacy from their families still wanted them involved in some way.
Lever Taylor et al. (2020) A qualitative investigation of models of community mental health care for women with perinatal mental health problems UK	Explore women’s experiences of specialist perinatal versus generic non-perinatal community mental health support	Qualitative	Semi-structured interviews	Thirty-six women. Mean age 33 years, (range under 25 to 40+ years). Primary Diagnosis depression (33%), bipolar affective disorder/psychosis/schizophrenia (33%).	Thematic analysis guided by Braun and Clarke (2006)	Women valued the specialist expertise offered by perinatal teams. Continuity of clinician was important to women. Inadequate resources and limited family involvement were reported.
Myors et al. (2014a) ‘My special time’: Australian women's experiences of accessing a specialist perinatal and infant mental health service Australia	Report on women’s experiences of accessing the support of specialist PIMH services	Qualitative study, part of a larger mixed-methods study exploring two PIMH services in Australia	Face-to-face or telephone interviews	Eleven women. Mean age 30.2 years, (range 20 to 39 years). Primiparas (9%), multiparas (91%). Previous PIMH client (27.2%).	Thematic analysis guided by Green et al. (2007)	Women reported a positive experience of the service, their relationship with clinician being a key component.
Myors et al*.* (2014b) Therapeutic Interventions in Perinatal and Infant Mental Health Services: A mixed methods Inquiry Australia	To explore the characteristics of women referred to specialist PIMH service and the therapeutic interventions used	Mixed methods	Face-to-face or telephone interviews and medical record data	Eleven women. Demographics reported in Myors (2014a).	Content analysis and thematic analysis guided by Braun and Clarke (2006) and Green et al. (2007)	The relationship built with clinicians was key to women’s experience of interventions.
Myors et al. (2015a) A mixed methods study of collaboration between perinatal and infant mental health clinicians and other service providers: Do they sit in silos? Australia	Report the collaborative practices between PIMH clinicians and other service providers from the perspectives of clinicians, managers, key stakeholders, and women service-users	Mixed methods	Semi structured interviews and medical record data	Eleven women participated in interviews. Demographics reported in Myors (2014a).	Content analysis and thematic analysis guided by Braun and Clarke (2006) and Green et al. (2007)	Women experienced negative consequences when collaboration between services was not effective.
Myors et al*.* (2015b) Engaging women at risk for poor perinatal mental health outcomes: A mixed-methods study Australia	Examine characteristics of women who engage in PIMH services and what factors are perceived to enhance or disrupt engagement with specialist PIMH services	Mixed methods	Semi structured interviews and medical record data	Eleven women participated in interviews. Demographics reported in Myors (2014a).	Thematic analysis guided by Braun and Clarke (2006)	Services that were flexible were positively experienced. Women felt more comfortable being assessed in their own homes. Women reported that they would have preferred a longer service.
Pilav et al. (2022) Experiences of Perinatal Mental Health Care among Minority Ethnic Women during the COVID-19 Pandemic in London: A Qualitative Study UK	Explore minority ethnic women’s experiences of perinatal mental health services during COVID-19 in London	Qualitative design	Semi structured interviews	Eighteen women. Mean age 33.4 years, (range 19 to 46 years). Primiparas (33.3%), multiparas (66.7%). Primary diagnosis depression (33.3%).	Thematic analysis guided by Braun and Clarke (2006)	Difficulties and disruptions to access during COVID 19 restrictions were felt emotionally by women. Both advantages and disadvantages were reported in relation to remote care.
Powell et al. (2020) Mothers’ experiences of acute perinatal mental health services in England and Wales: a qualitative analysis UK	Explore women’s views and experiences of generic wards, MBUs, and crisis resolution teams	Qualitative design that forms part of a quasi-experimental study	Analysis of the free-text comments from a s service-user designed survey	One hundred and thirty-nine women. Age range 16 to 49 years. Primiparas (55.4%), multiparas (44.6%). Married or cohabiting n=111 (79.9%).	Thematic analysis guided by Braun and Clarke (2006,2014)	Two themes identified: support networks and staff authority. Mothers reported the benefits of positive non coercive relationships with family and staff for their recovery.
Viveiros and Darling (2018) Barriers and facilitators of accessing perinatal mental health services: The perspectives of women receiving continuity of care midwifery Canada	Explore access to PMH care and identify barriers and facilitators to accessing PMH services	Qualitative descriptive design	Semi-structured interviews and focus groups	Sixteen women. Demographics not reported.	Thematic analysis guided by Braun and Clarke (2006)	Inadequate capacity resulted in long waiting lists and inequity of access. Services located far from women’s homes were inaccessible. Flexible services facilitated access and were found to be very helpful.
Wright et al. (2018) Patient experience of a psychiatric Mother Baby Unit New Zealand	Explore mothers’ experiences of MBU service providing evidence to inform health policy	Exploratory mixed methods	Semi-structured interviews, anonymous written feedback, and verbal feedback	Forty-five women. Mean age 32.4 years, (range 18 to 42 years). Primiparas (62.2%), multiparas (37.8%). Primary diagnosis depression (34.4%).	Thematic analysis conducted in an experiential, realist framework	Service strengths included co-admission of mother and infant, staff warmth and availability, inclusion of families, and transparent practice.

*Only qualitative data relevant to the review question was extracted from the included studies

## GRADE CER-Qual

To assess confidence in the synthesized qualitative review findings the GRADE-CERQual approach was used (Lewin et al. 2018). This involved assessment of methodological limitations of the individual studies contributing to a review finding; the relevance to the review question of the studies contributing to a review finding; the coherence of the review finding; and the adequacy of data supporting a review finding (Noyes et al. 2018). A CERQual summary of qualitative evidence table was developed using the Interactive Summary of Qualitative Findings (iSoQ) online tool (Table 3).

**Table 3:** Grade-CERQual assessment

**#**	Summarised review finding	GRADE-CERQual Assessment of confidence	Explanation of GRADE-CERQual Assessment	References
**THEME 1: CONNECTED RELATIONSHIPS**				
1	Participants valued personal attributes of clinicians such as sensitivity, warmth, and dedication.	High confidence	No/Very minor concerns regarding methodological limitations, No/Very minor concerns regarding coherence, Minor concerns regarding adequacy, and Minor concerns regarding relevance	Griffiths et al. 2019; Coates et al. 2017; Myors et al. 2014b; Wright et al. 2018; Viveiros & Darling 2018;
2	Having a non-judgmental clinician was important and comforting to women	High confidence	Minor concerns regarding methodological limitations, Minor concerns regarding coherence, No/Very minor concerns regarding adequacy, and Minor concerns regarding relevance	Griffiths et al. 2019; Hauck et al. 2013; Higgins et al. 2016; Coates et al. 2017; Lever Taylor et al. 2020; Myors et al. 2015b; Wright et al. 2018; Bruce & Hackett 2021;
3	Peer relationships supported women’s recovery in group therapy sessions and on MBUs	Moderate confidence	Minor concerns regarding methodological limitations, No/Very minor concerns regarding coherence, Minor concerns regarding adequacy, and Minor concerns regarding relevance	Griffiths et al. 2019; Greaves et al. 2021; Powell et al. 2020; Viveiros & Darling 2018;
4	Women reported reduced contact and difficulty accessing family support due to clashes with relatives' work, long travel distances, inflexible visiting hours, and childcare commitments.	Moderate confidence	Minor concerns regarding methodological limitations, No/Very minor concerns regarding coherence, Moderate concerns regarding adequacy, and Minor concerns regarding relevance	Griffiths et al. 2019; Lever Taylor et al. 2019; Powell et al. 2020;
5	Women reported that their families were excluded from services.	Low confidence	Minor concerns regarding methodological limitations, Minor concerns regarding coherence, Moderate concerns regarding adequacy, and Minor concerns regarding relevance	Lever Taylor et al. 2019; Lever Taylor et al. 2020;
**THEME 2: NEW BEGINNINGS**				
6	Specialist PMHS supported women in overcoming past traumas which brought positive changes to their lives	High confidence	Minor concerns regarding methodological limitations, No/Very minor concerns regarding coherence, Moderate concerns regarding adequacy, and Minor concerns regarding relevance	Coates et al. 2017; Myors et al. 2014b; Wright et al. 2018; Bruce & Hackett 2021;
7	Services helped women to grow in confidence as a mother.	Moderate confidence	Minor concerns regarding methodological limitations, Minor concerns regarding coherence, Moderate concerns regarding adequacy, and Minor concerns regarding relevance	Greaves et al. 2021; Coates et al. 2017; Myors et al. 2014b; Wright et al. 2018;
**THEME 3: MEANINGFUL SERVICE DELIVERY**				
8	Service flexibility facilitated women's engagement with services.	High confidence	Minor concerns regarding methodological limitations, Minor concerns regarding coherence, Minor concerns regarding adequacy, and Minor concerns regarding relevance	Griffiths et al. 2019; Coates et al. 2017; Lever Taylor et al. 2020; Myors et al. 2015b; Viveiros & Darling 2018;
9	Continuity of clinician was important to women's experience of services.	High confidence	Minor concerns regarding methodological limitations, No/Very minor concerns regarding coherence, Minor concerns regarding adequacy, and Minor concerns regarding relevance	Griffiths et al. 2019; Hauck et al. 2013; Higgins et al. 2016; Lever Taylor et al. 2020; Myors et al. 2014b; Viveiros & Darling 2018;
10	Information regarding medication management was an important aspect of care to women	Moderate confidence	Minor concerns regarding methodological limitations, No/Very minor concerns regarding coherence, Moderate concerns regarding adequacy, and Minor concerns regarding relevance	Lever Taylor et al. 2019; Higgins et al. 2016; Lever Taylor et al. 2020; Wright et al. 2018;
11	Poor communication and continuity between services were difficulties experienced by women post discharge.	Low confidence	Minor concerns regarding methodological limitations, Minor concerns regarding coherence, Moderate concerns regarding adequacy, and Minor concerns regarding relevance	Griffiths et al. 2019; Lever Taylor et al. 2020;
12	Women reported a lack of discharge supports following MBU inpatient treatment.	Low confidence	No/Very minor concerns regarding methodological limitations, No/Very minor concerns regarding coherence, Moderate concerns regarding adequacy, and Minor concerns regarding relevance	Griffiths et al. 2019; Lever Taylor et al. 2020;

## Themes

Thematic synthesis distinguished three themes reflecting different concepts of women’s experiences across services: 1) connected relationships, 2) new beginnings, 3) impactful service provision. Themes and subthemes are presented in a thematic map (Supplementary File 4). Direct participant quotes supporting the three themes is illustrated in Table 4.

**Table 4:** Thematic structure with illustrate quotes

Theme	Illustrate quotes
Theme 1: Connected relationships 1.1: Relationships with clinician	‘Some staff made an effort to really understand me…the staff [MBU] were very astute at reading my struggling signals’ (Powell *et al.* 2020, p.5 & 6). ‘She came up after the birth…and she sat with me… I was very lucky. The service I got was fantastic’ (Higgins *et al*. 2016, p.32). ‘She was a very gentle person. It was a kind of softness, it wasn’t harsh … she listened, and she asked … the right questions … there was something in her approach that made me feel that I could open up to her… and feel comfortable’ (Myors *et al.* 2014a, p.273). ‘[The clinician] would listen but he wouldn’t pass judgement’ (Coates *et al*. 2017, p.93). ‘She went through the hospital system and did a complaint for me… no one responded to her or myself, but the fact that she was proactive and really tried to help seek justice in the situation… that helped me emotionally’ (Myors *et al.* 2014b, p.377). ‘[I] really valued having clinical staff available all the time, it really helped me feel safe and that I could trust enough to relax and recover’ (Wright *et al.* 2018, p.8). ‘You could definitely see the ones [on the MBU] that were, you know, a bit more under stress and a bit more snappy..’ (Griffiths *et al.* 2019, p.8).
1.2: Family involvement in care	‘When I went into the mother and baby [unit]… we had support as a family. All of us… They worked with us as a family as opposed to an individual’ (Griffiths *et al.* 2019, p.7). ‘My mother and I now have a plan if I become unwell again, to manage my situation, now that we know more’ (Wright *et al*. 2018, p.9). ‘The psychologist at the MBU suggested that my partner comes along to one of the sessions. But he just couldn’t find time for it, basically, and to do his work…it’s a few hours travelling’ (Griffiths *et al.* 2019, p.7). ‘There was sometimes 15 mins left after which he would be asked to leave’ (Powell *et al.* 2020, p.5). ‘[The community mental health team] haven’t told me ‘how would you like us to involve [your husband]? They just told me to bring him to appointments. But I didn’t want to bring him to my appointments because they might bring up something that he doesn’t know’ (Lever Taylor *et al.* 2019, p.9).
1.3: Peer support	‘Other mothers [were] vital in my recovery’ (Powell *et al*. 2020, p.6). ‘I think you never really know what people are going through, but I think if you’re in a group with other mums with mental health problems, I think that was helpful to me to sort of see them having a relationship with their babies’ (Greaves *et al.* 2021, p.179). ‘Just knowing that there were other mums, it was just like the biggest comfort ever. I just felt like, oh my gosh, I’m not the only one’ (Griffiths *et al.* 2019, p.8). ‘I think that would have been helpful for me in terms of getting out of the house and speaking with people who could really understand what I was going through because my husband is super supportive, but he doesn't get it fully’ (Viveiros and Darling 2018, p.12).
Theme 2: New beginnings 2.1: Dealing with the past	‘I didn’t realise myself but that a lot of my issues are from… past experiences and childhood and all that sort of stuff’ (Myors *et al.* 2014b, p.381). ‘I’ve had in a way a pretty traumatic past, so it was being able to talk to somebody and for them to actually understand and not criticise me was the support I needed’ (Coates *et al*. 2017, p.94).
2.2 Gaining new insights	‘Once I began to feel better, I was able to learn to see my child’s needs, what my anxiety meant to his experience. It was really hard, but it’s the mother I want to be’ (Wright *et al.* 2018, p.9). ‘I was supported to work through things myself’ (Coates *et al.* 2017, p.93).
Theme 3: Meaningful service delivery 3.1: Organisation of services	‘It was very flexible, depending on me too, sometimes I wasn't very good at going out, and they would (fit) in with that, but the home service was the best, that they could come here’ (Myors *et al.* 2015b, p.246). ‘I just think [my perinatal mental health nurse] was massively reassuring… you have a troop of miscellaneous midwives, it’s a different one each time… so [my perinatal nurse] was like a constant... a sort of counterpoint to all of that’ (Lever Taylor *et al.* 2020, p.4). ‘…because of the distance, it was really difficult to access, so I feel like more women would go and access these services if they were closer to them’ (Viveiros and Darling 2018, p.12). ‘Having baby on the unit too is such a big healer, just on its own’ (Wright *et al.* 2018, p.9). ‘I haven’t attended any groups because I’m not confident to do it over the phone. It would have been easier for me in person but because of COVID, I told my nurse that I’m not confident doing these calls with other people over the phone’ (Pilav *et al.* 2022, p.7).
3.2 Therapeutic interventions	‘to talk to her about things, it was safe, it just felt really safe and comfortable to open up’ (Myors *et al.* 2014a, p.273). ‘My mood swings aren’t as intense or frequent because I used the tools that I learnt, so that’s going to have a positive impact on Daisy. Her environment is calmer at home’ (Greaves *et al.* 2021, p.178).
3.3 Care at discharge	‘There needs to be a lot more information provided about…what [is] offered in the community’(Viveiros and Darling 2018, p.10). ‘What was the point of going to [an MBU] if it doesn’t get followed up at all? I still don’t understand’ (Griffiths *et al.* 2019, p.10). ‘…what do you do with someone who still needs your help? … for some people who don’t need help, their baby’s born and they’re coping ok and that’s good … but for people that turn out to be a more long-term issue, there’s nothing’ (Myors *et al*. 2014a, p.273). ‘They tried to transition [me] into another service, and that has not been successful … not because of me, but because the other service just … keeps forgetting … It’s very disappointing to me …you get to a point now when they haven’t called you … three times when they said they would, … I don’t want to put myself out and call. I’m not comfortable with that now’ (Myors *et al.* 2015a, p.10).

### Theme 1: Connected relationships

This theme reports on various interpersonal relationships which were comprehensively described by women and significant to how care was experienced from specialist PMHS. Fourteen of the sixteen included studies contributed to this theme which is reported in three subthemes: relationships with clinicians; family involvement in care; and peer support.

#### Subtheme 1.1 Relationship with clinicians

Relationships with PMHS clinicians was reflected in twelve papers and frequently outlined as being fundamental to women’s experience of care. The relationship with clinicians facilitated personal change in the women’s lives. Women valued being treated like ordinary mothers and felt clinicians had real insight and understanding of PMH conditions (Coates et al. 2017; Lever Taylor et al. 2020; Powell et al. 2020).

Women trusted their perinatal teams who they described as professional, dedicated, and like part of their family (Hauck et al. 2013; Coates et al. 2017; Griffiths et al. 2019; Lever Taylor et al. 2020). Participants valued personal attributes of clinical staff highlighting these as meaningful to positive service experience. Sensitivity, warmth, consistency, and encouragement from clinicians helped women rely upon staff for support in working towards their goals (Myors et al. 2014a; Coates et al. 2017; Viveiros and Darling 2018; Wright et al. 2018; Griffiths et al. 2019). In eight of the included studies women perceived clinicians as being non-judgemental which was viewed as important and comforting (Hauck et al. 2013; Myors et al. 2015b; Higgins et al. 2016; Coates et al. 2017; Wright et al. 2018; Griffiths et al. 2019; Lever Taylor et al. 2020; Bruce and Hackett 2021). However, several women expressed concerns about being judged (Myors et al. 2015b), and were initially distrustful of staff (Griffiths et al. 2019).

Clinicians acted as advocates on the women’s behalf ensuring their needs were met and that they were connected to required services (Myors et al. 2014a; Higgins et al. 2016; Viveiros and Darling 2018; Lever Taylor et al. 2020). Advocacy was important to women who felt they could not always speak up for themselves, especially when talking to other professionals. The presence and availability of staff was reported as central to women’s recovery in four studies (Hauck et al. 2013; Wright et al. 2018; Lever Taylor et al. 2020; Powell et al. 2020). Perceived understaffing or reluctance to help was heavily criticised by women in the MBU setting (Wright et al. 2018; Griffiths et al. 2019; Powell et al. 2020). Staff seen as being unavailable were found to be unempathetic and not proactive enough by women, reducing necessary care and support (Wright et al. 2018; Griffiths et al. 2019; Powell et al. 2020).

#### Subtheme 1.2 Family involvement in care

Five studies described women’s perceptions of how services worked with their families (Wright et al. 2018; Griffiths et al. 2019; Lever Taylor et al. 2019; Lever Taylor et al. 2020; Powell et al. 2020). These represented MBU services, specialist community PMHS, and NHS (UK) specialist PMHS. Women recognised that their illness affected their whole family and valued family involvement in their care (Wright et al. 2018; Powell et al. 2020).

Partners and family being welcomed into the MBU setting was important to women (Griffiths et al. 2019; Lever Taylor et al. 2019; Powell et al. 2020). Family inclusion was facilitated in various ways including being kept well-informed; regular invitations to ward rounds; encouraging contribution of their own observations; and taking their views into account (Griffiths et al. 2019; Lever Taylor et al. 2019). Family involvement allowed for improved communication between women and their families and joint planning for the future (Wright et al. 2018).

Women reported reduced contact and difficulty accessing family support due to clashes with relatives’ work, long travel distances, inflexible visiting hours, and childcare commitments (Griffiths et al. 2019; Lever Taylor et al. 2019; Powell et al. 2020). Providing private family rooms and extending visiting hours accommodated families’ schedules where distances to the MBU were long (Griffiths et al. 2019). Experiences of families being excluded by services was also reported (Lever Taylor et al. 2019; Lever Taylor et al. 2020). Exclusion was reported in various ways including not being provided with adequate information; having few opportunities to be involved in appointments or treatment decisions; and a lack of acknowledgement of family members own distress (Lever Taylor et al. 2019). Women believed supports for their family members should be available (Lever Taylor et al. 2019; Powell et al. 2020).

Adding to the complexity, some women reported that too much family involvement could leave them feeling marginalised, deprioritised, or reduce their protected time with clinicians (Lever Taylor et al. 2019). Concerns that family involvement could affect their autonomy or place unmanageable demands on them were also reported (Lever Taylor et al. 2019). Women who wished for privacy still wanted their family involved in some way, desiring clinician’s help in negotiating family inclusion given their individual circumstances.

#### Subtheme 1.3 Peer support

Peer relationships promoted women’s recovery in both group therapy settings (Viveiros and Darling 2018; Greaves et al. 2021), and on MBUs (Griffiths et al. 2019; Powell et al. 2020). Women felt they could relate to other mothers with similar experiences, supporting and helping each other in a non-judgemental way (Griffiths et al. 2019; Powell et al. 2020). Women appreciated being admitted to the MBU alongside other mothers experiencing PMH conditions as they often had the same worries and could bond and support each other (Griffiths et al. 2019; Greaves et al. 2021).

A sense of community was formed in the MBU and group therapy setting which instilled hope (Viveiros and Darling 2018; Griffiths et al. 2019). In some cases, friendships were long lasting, providing mothers with continued social support by peers (Griffiths et al. 2019). As opposed to online or telephone support in-person groups gave women valuable contact from others at a time they could otherwise be incredibly lonely (Viveiros and Darling 2018). When MBU staff discouraged women from talking to other mothers’ women felt this limited their supportive relationships and possibly damaged relationships with staff (Powell et al. 2020).

### Theme 2: New beginnings

This second identified theme is reported under the subthemes of dealing with the past and gaining new insights. This theme captures how women moved forward into a place of increased confidence and understanding. Findings from eight of the included studies contributed to this theme.

#### Subtheme 2.1 Dealing with the past

Women described how specialist PMHS supported them in overcoming past traumas which brought positive changes to their lives (Myors et al. 2014b; Coates et al. 2017; Wright et al. 2018; Bruce and Hackett 2021). Many women had not spoken about their trauma prior to attending services and used this time to make sense of difficult childhood memories and become emotionally available for their child (Coates et al. 2017; Bruce and Hackett 2021). Art therapy was found to support women in making connections between their created images and feelings of loss and shame experienced as a child (Bruce and Hackett 2021).

#### Subtheme 2.2 Gaining new insights

Women recognised that their ability to understand their infant’s needs, and develop a mother-infant bond, had been impaired by their experience of perinatal mental illness (Wright et al. 2018; Lever Taylor et al. 2020; Bruce and Hackett 2021). Women explored these concerns and worked to strengthen the mother-infant bond through therapeutic work (Wright et al. 2018; Griffiths et al. 2019; Bruce and Hackett 2021).

Across four studies women reported that services helped them grow in confidence as a mother which enhanced their sense of wellbeing (Myors et al. 2014a; Coates et al. 2017; Wright et al. 2018; Greaves et al. 2021). Increased confidence in their parenting ability reduced women’s anxiety and helped them feel more connected to their baby (Myors et al. 2014a; Greaves et al. 2021). Women began to think critically about their situation and work towards their own solutions with a sense of empowerment and self-understanding (Coates et al. 2017; Wright et al. 2018; Bruce and Hackett 2021).

### Theme 3: Meaningful service delivery

Theme three meaningful service delivery reports on data from fifteen of the included studies under the subthemes of organisation of services; treatment interventions; and care at discharge. This theme focuses on women’s experiences of how services are delivered and their perspectives on treatment interventions.

#### Subtheme 3.1 Organisation of services

Service flexibility facilitated women’s engagement with services and was reported as an essential aspect of service delivery across five papers (Myors et al. 2015b; Coates et al. 2017; Viveiros and Darling 2018; Griffiths et al. 2019; Lever Taylor et al. 2020). Arrangement of appointments to suit family schedules and home visits were appreciated and valued by women (Coates et al. 2017; Viveiros and Darling 2018; Griffiths et al. 2019; Lever Taylor et al. 2020).

Continuity of clinician was significant to women’s service experience and reported across six papers (Hauck et al. 2013; Myors et al. 2014a; Higgins et al. 2016; Viveiros and Darling 2018; Griffiths et al. 2019; Lever Taylor et al. 2020). Women did not feel comfortable repeating their story with unfamiliar clinicians. Continuity allowed women to build ongoing relationships and provided feelings of safety, comfort, and dependability (Hauck et al. 2013; Myors et al. 2014a; Higgins et al. 2016; Viveiros and Darling 2018; Lever Taylor et al. 2020).

Difficulty accessing services was identified by women as a barrier to receiving required care. Women reported limited provision of MBU beds, long distances to services, long waiting lists, difficulties traveling with their new-born, and not being permitted to bring their baby to appointments as challenges to accessing services (Viveiros and Darling 2018; Griffiths et al. 2019; Lever Taylor et al. 2020; Greaves et al. 2021; Pilav et al. 2022).

Some women identified having their baby present during specialist group DBT sessions as being a distraction or impediment to learning, however most women felt positive about having their baby present believing their baby regulated their emotions and benefitted from the social experience (Greaves et al. 2021). Groups were more accessible to women when they could bring their baby as this eliminated practical considerations such as childcare and maintaining breastfeeding (Greaves et al. 2021). Having their baby present during MBU admission was reported as positive to women’s recovery (Wright et al. 2018; Griffiths et al. 2019).

Greater accessibility and ease were experienced when appointments were provided remotely or through home visits (Myors et al. 2015b; Coates et al. 2017; Viveiros and Darling 2018; Pilav et al. 2022). Women reported the benefits of home visits and remote services when face-to-face contact was less necessary or difficult to access. However, diverse feelings relating to remote contact were also reported. Appointments at the service centre were preferred when women felt they could not talk openly at home (Myors et al. 2015b), others reported logistical difficulties encountered with virtual meetings (Pilav et al. 2022). Movement of groups to a remote platform due to COVID-19 procedures left some women feeling anxious as care decisions felt out of their control.

#### Subtheme 3.2 Therapeutic interventions

Across six papers women reported that clinicians, service environments, and therapeutic interventions felt like a safe space in which they could focus on their recovery (Myors et al. 2014a; Coates et al. 2017; Griffiths et al. 2019; Powell et al. 2020; Bruce and Hackett 2021; Greaves et al. 2021).

Various beneficial treatment interventions were reported which included grounding techniques (Coates et al. 2017); dialectical behavioural therapy skills (Greaves et al. 2021); specialist art therapy (Bruce and Hackett 2021): mindfulness and Cognitive Behavioural Therapy (CBT) (Wright et al. 2018); individual counselling (Viveiros and Darling 2018); parent-infant therapeutic work (Wright et al. 2018); and support work with clinicians (Myors et al. 2014b; Myors et al. 2015b; Higgins et al. 2016; Coates et al. 2017; Viveiros and Darling 2018).

Informational support particularly in relation to medication management was an important aspect of care for women (Higgins et al. 2016; Wright et al. 2018; Lever Taylor et al. 2020). Several women questioned the use of pharmacological treatment in their care, wanting more information and explanation about the potential risks and benefits (Wright et al. 2018; Lever Taylor et al. 2019). Where women felt pressure from health providers to take medication this was negatively experienced (Viveiros and Darling 2018; Powell et al. 2020). Insufficient information about available services were described as a barrier to accessing care (Viveiros and Darling 2018).

Women sought various interventions which were either unavailable or difficult to access due to waiting lists. These included psychological interventions (Lever Taylor et al. 2020); couples/family therapy (Lever Taylor et al. 2019); and non-pharmacological activities on MBU (Wright et al. 2018). Wanting greater practical supports with infant care was reported in three studies (Wright et al. 2018; Griffiths et al. 2019; Lever Taylor et al. 2020).

#### Subtheme 3.3 Care at discharge

Experiences of discharge from specialist PMHS was reported across four studies (Myors et al. 2014a; Myors et al. 2015b; Griffiths et al. 2019; Lever Taylor et al. 2020). Following MBU admission women wanted help continuing their recovery into the community but described a lack of accessible supports (Griffiths et al. 2019; Lever Taylor et al. 2020). Women who initiated their discharge from services felt confident that they would remain well (Myors et al. 2014a). However, discharge was a stressful experience when women felt they weren’t ready resulting in significant anxiety (Myors et al. 2014a). Women were aware that discharge was a timed decision with services generally supporting women until their child’s first birthday. Difficulties associated with post discharge care included: poor communication and continuity between services; inadequate information provision; long waiting lists; no follow up from referral service; and no consistent point of contact (Myors et al. 2014a; Griffiths et al. 2019).

## Discussion

Despite the variety of settings and specialist PMHS explored in this review common themes were identified across the studies. Findings illustrate the meaningfulness of relationships to women attending specialist PMHS. Women valued clinicians who were insightful, understanding, and non-judgemental. Peer support from other mothers attending services provided women comfort and hope. Family involvement in care was important to many women. Service intervention enabled women to grow in confidence as mothers and gain new insights into their infant’s needs. Service-related factors which facilitated women’s attendance of services included service accessibility, flexibility, and provision of continuity of care.

Theme one which highlights the importance and beneficial nature of interpersonal relationships built with staff was reported as fundamental to women’s attendance and experience of services. This is consistent with the literature which identifies the positive impacts of non-judgemental emotional support from staff to women with PMH conditions (Biggs et al. 2015; Ford et al. 2019). A study exploring ethnic minority and migrant women’s experiences of accessing PMHS by Soltani et al. (2021) support the need to provide a safe space for women to talk and be really listened to. Compassionate non-judgemental care fosters hope and optimism in perinatal women about their mental health treatment (Megnin-Viggars et al. 2015).

Findings of this review identify that women value family involvement in their care and treatment decisions. Women seek supportive interventions to be provided to their family members which were not always available. Inflexible service structures such as restrictive visiting times were perceived as challenging to women and limited their contact with family members in the MBU setting. The Centre of Perinatal Excellence (COPE) Clinical Practice Guideline (COPE 2017) emphasise the need to take a family-centred approach when working with women with PMH conditions, working in partnership with women and their significant others, and valuing the importance of clearly communicated information. Converse findings of this review suggest that several women experience concerns about family involvement in their care however only a small number of studies contributed to this finding. Further qualitative research among perinatal women and clinicians is required to explore factors which impact women’s preferences towards family involvement. Screening is required to identify women experiencing difficulties in their domestic relationships to determine an individualised plan with suitable family involvement and required supports (Maybery et al. 2021).

Peer relationships developed between mothers attending services were perceived as positive to women’s service experience and offered them a valued sense of community and support. Peer support as an intervention strategy has the potential to address some of the key concerns associated with poor PMH such as isolation and poor social and emotional supports (Biggs et al. 2019; Rice et al. 2022). A systematic review and meta-analysis found that peer support interventions were well received by women in the perinatal period and may offer a cost-effective strategy to address the shortage of mental health care providers (Huang et al. 2020). The de-stigmatising and flexible focus of peer support was highly valued, reducing women’s sense of isolation and offering a sense of hope (Rice et al. 2022). Further research has been recommended to examine the effectiveness and consistency of peer support interventions, whilst improving mothers’ engagement with formal treatment services.

Continuity of care is widely acknowledged in the literature and strategic policies as being central to both perinatally focused and generic quality mental health care (Sweeney et al. 2016; Weaver et al. 2017). A systematic review exploring barriers to implementing perinatal mental healthcare established the importance of continuity of care across the perinatal care pathway (Webb et al. 2021). This study found one of the most cited barriers in the provision of optimal PMH care to be a lack of continuity of care. This supports the finding of this review which identifies continuity of care and continuity of clinician as being significant in facilitating women’s attendance at specialist PMHS.

The second theme of this review found that specialist PMHS supported women in overcoming past traumas and facilitated women to develop new insights into their child’s needs which strengthened their mother-infant bond. A study carried out in a mother and baby day hospital in the US reported high prevalence rates of trauma-related disorders (20.6%) as a primary diagnosis among attending perinatal women (Kim et al*.* 2021). This study in addition to wider literature recommends a trauma-informed approach to guide PMH care with the need for increased research focus on trauma sensitive interventions (Granner and Seng 2021; Kim et al. 2021; Perera et al. 2023). Resources and supports available through specialist PMHS to perinatal women from a trauma-informed lens may help alleviate the lasting impact of trauma and related psychological symptoms (Perera et al. 2023).

Recently published guidelines by the World Health Organisation (WHO, 2022) put forward guidance for the monitoring and evaluation of PMHS. These guidelines focus on determining how well PMHS are being delivered and identify effective referrals as an essential service provision. International guidelines recommend that women with PMH conditions have access to local specialist PMHS ensuring their condition is assessed and monitored appropriately, with access to the most suitable treatments (National Institute for Health and Care Excellence (NICE) 2014; Health Service Executive (HSE) 2017; Centre of Perinatal Excellence (COPE) 2017). Findings of this review suggest that referral processes between perinatal services do not always take place in a coordinated way with non-follow up being reported in some instances which negatively influenced women’s perception of services. Effective referral systems with clear mechanisms and care pathways are required to provide women with links to required services (WHO, 2022). Women’s trust in services may be damaged and expectations of services reduced when referral processes are interrupted, impacting their decision to engage with services in the future (WHO, 2022).

Discharge from perinatal services was identified as challenging and anxiety provoking to women who felt they still required service supports (Myors et al. 2014a). However, a limited number of included studies reported on women’s experience surrounding their discharge from specialist PMHS and this was not the primary focus of any papers. Further investigation is needed to inform services on how best to identify women in need of additional supports and the risks associated with discharge as a timed decision. Howard and Khalifeh (2020) call for further research to examine whether extension of services to the second year after birth is beneficial to some women’s outcomes and feasible to service capacity and cost.

## Strengths and Limitations

A rigorous and systematic approach was used in this QES to explore women’s experiences of specialist PMHS. Five countries are represented in this review with the origin of studies reflecting where PMHS have been developed. As a result, the findings of this study may lack transferability to other contexts and regions. Consistent themes emerged from across the various specialist settings and interventions represented in this QES. Although the breath of services examined allowed for a wide range of women’s experiences to be synthesised this may have limited the depth of understanding in specific perinatal contexts. Among the included studies which reported the socioeconomic status of women married and cohabiting women represented typically 80% of study participants therefore limiting representation of women who may have additional PMH risk factors and unique service experiences. This review recommends that a diverse sample of women be used in future exploration of PMHS experience.

## Implications for future research

The evidence of this QES highlights various areas which require further research. Women’s views, experiences, and outcomes of pre-conception specialist care were absent. Outside of one study exploring remote service delivery during COVID-19 restrictions and limited data on home visits, focused analysis on women’s perceptions of remote PMHS delivery and telehealth are limited. Whilst this review found evidence of women’s desire for increased family involvement in care various individual concerns were reported which warrant further qualitative exploration to determine how best to meet women’s individual needs. Further qualitative evidence is also required to understand challenges women experience when being discharged from or transferred between perinatal services. With maternal mortality rates being a significant concern in the perinatal period, it is important to identify and plan for women’s continuing care needs post discharge from specialist services, ensuring effective collaborative care planning practices are in place to enable women and their families to build a positive future. Future research is also required to capture women’s experiences of newly established specialist PMHS e.g.: Ireland which will contribute to existing knowledge.

## Conclusion

In conclusion, women seek provision of continuity of care from supportive services which help them feel understood in a non-judgemental way. Greater provision of accessible and flexible services including access to local treatment intervention options would promote women’s attendance at services and positively impact health outcomes. It is evident that there is a need to consider women’s individual psychosocial circumstances, and preferences surrounding family inclusion, remote care, and discharge needs.
